# Production of cinnamates and benzoates glucose esters by bioconversion using *Escherichia coli* expressing a glucosyltransferase from sweet potato

**DOI:** 10.5511/plantbiotechnology.24.0705a

**Published:** 2024-09-25

**Authors:** Yuki Kobayashi, Nasanjargal Dorjjugder, Goro Taguchi

**Affiliations:** 1 Department of Applied Biology, Faculty of Textile Science and Technology, Shinshu University, Ueda, Nagano 386-8567, Japan

**Keywords:** bioconversion, ester-forming glycosyltransferase, sweet potato, whole-cell biocatalyst

## Abstract

Sweet potatoes (*Ipomoea batatas*) produce glucose esters from cinnamates during stress responses. We isolated a glucosyltransferase, *IbGT1* (UGT84A20), from sweet potato and characterized its recombinant enzyme heterologously expressed in *Escherichia coli*. The recombinant IbGT1 enzyme reacted with cinnamates, especially *p*-coumaric acid, ferulic acid, and sinapic acid, and showed activity against benzoates with an optimum pH of 5.0–5.5. The enzyme also reacted with flavonoids under alkaline conditions (pH 8.0), with the main product being 7-*O*-glucosides. Bioconversion using *E. coli* expressing IbGT1 (Ec-IbGT1) as a whole cell biocatalyst converted 200 µM cinnamates such as *p*-coumaric acid and benzoates such as *p*-hydroxybenzoic acid into their glucose esters mostly within 3–6 h. The conversion rates for caffeic acid and sinapic acid were much lower than those for the other substrates tested, which took more than 6 h, despite the enzymatic activities of IbGT1 against these compounds being comparable to the others. Scaled-up production combined with repeated administration of sinapic acid, *trans*-cinnamic acid and *p*-hydroxybenzoic acid at a concentration of 200 µM yielded their glucose esters with conversion rates of 74–95% in 20–30 h. These results suggest that the Ec-IbGT1 system efficiently produces these glucose esters.

## Introduction

Plants produce many specialized metabolites to cope with environmental conditions and accumulate many of these metabolites as glycosides. In plants, most of the enzymes involved in glycosylation reactions of low-molecular compounds are uridine 5′-diphosphate (UDP)-sugar dependent glycosyltransferases (UGTs), those produce several glycosides, including *O*-, *N*-, *S*-, *C*- and carboxy-glycosides (glycose ester) (Yonekura-Sakakibara and Hanada 2011). Generally, glycosylation increases the water solubility and chemical stability of compounds; therefore, it is considered to play an important role in the accumulation of compounds and regulation of reactivity (Bowles et al. 2005). Glycosylation is also important for the utilization of chemical compounds, because it alters their physical properties and bioactivities. Chemical synthesis of these glycosides has been performed; however, it is often difficult to synthesize phenolics because they have multiple reactive groups that can be glycosylated. Therefore, enzymatic synthesis is advantageous for the production of these glycosides, as they are substrate-specific and regiospecific (De Bruyn et al. 2015). To overcome the high cost of UDP-sugars, several methods have been proposed, such as the regeneration of UDP-sugars using sucrose synthase (Masada et al. 2007), transglycosylation reactions using inexpensive glycosides or glycans as sugar donors instead of UDP-sugars (Chen et al. 2016). Whole-cell biocatalysis is an efficient way to produce glycosides, in which a sugar donor is supplied by the host (De Bruyn et al. 2015; Lim 2005) when substrate uptake is possible. In recent years, many phytochemicals have been produced de novo in microbial cells using a synthetic biology approach, and whole-cell biocatalysts are still effective and convenient for the pinpoint conversion of substrates. For example, 2(5H) furanones, key flavors of caramel and roasted coffee, were successfully converted to their odorless glucosides using *E. coli* cells expressing UGT84A45 with a yield of 5.3–7.2 g l^−1^ (Effenberger et al. 2019). We have also successfully converted flavonoids into their 7-*O*-glucosides or *C*-glucosides using *E. coli* cells expressing regiospecific UGTs (Dorjjugder and Taguchi 2022; Dorjjugder et al. 2021; Ito et al. 2014).

Phenolic acids, including hydroxycinnamates and benzoates, are specialized metabolites found in various plant species. They have defensive functions, such as anti-UV and antimicrobial activities, and attractive functions through their color and flavor (Taofiq et al. 2017). They are particularly attractive because of their antioxidant and anti-inflammatory effects (Kylli et al. 2008). Phenolic acids often accumulate in plants as glycosides (*O*-glycosides or glucose esters), which increase water solubility. The glucose esters of phenolic acids (acyl-glucoses) function as substrates for biosynthetic enzymes such as serine carboxypeptidase-like acyltransferases or acyl-glucose-dependent glucosyltransferases, which form several acylated compounds or glucosides such as sinapoyl malate and anthocyanins (Sasaki et al. 2014; Stehle et al. 2009). They reported several bioactivities; for example, galloyl glucose and sinapoyl glucose showed antioxidant and anti-proliferative activities in cholangiocarcinoma cells (Suyanto et al. 2024). Therefore, these conjugates are important for their use as health-promoting substances; however, there are few reports on their enzymatic production of them (Han et al. 2016; Matsuba et al. 2008; Suyanto et al. 2024).

Sweet potato (*Ipomoea batatas*) is highly active in the biosynthesis of cinnamate derivatives, accumulating chlorogenic acid and related compounds in defense responses, such as wounding or herbivore attack (Chen et al. 2023; Kojima and Uritani 1973). It has also been reported to accumulate cinnamoyl glucose upon treatment with ^14^C-cinnamate (Kojima and Uritani 1973), and a glucosyltransferase catalyzing cinnamate glucose ester formation has been partially purified from sweet potatoes (Shimizu and Kojima 1984). Therefore, highly reactive enzymes that catalyze glucose ester formation from cinnamates may be present in sweet potatoes. In this study, we isolated the gene encoding this enzyme and tested it as a biocatalyst for the production of acyl-glucose.

## Materials and methods

### Chemicals

The following substrate and standard compounds were obtained: *trans*-cinnamic acid, *p*-coumaric acid, and *p*-hydroxybenzoic acid (Nacalai Tesque, Kyoto, Japan); *p*-methoxycinnamic acid, ferulic acid, *o*-coumaric acid, caffeic acid and sinapic acid (Sigma-Aldrich, St. Louis, MO, USA); *o*-hydroxybenzoic acid (salicylic acid), and benzoic acid (FUJIFILM Wako Pure Chemical, Osaka, Japan); kaempferol (Tokyo Chemical Industry, Tokyo, Japan); quercetin and quercetin 3-*O*-glucoside (Cayman Chemicals, Ann Arbor, MI, USA); kaempferol 3-*O*-glucoside and quercetin 4′-*O*-glucoside (Extrasynthese, Genay, France); and UDP-glucose (Carbosynth, Compton, Berkshire, UK). *p*-Coumaroyl glucose is a kind gift from Dr. Masayuki Nozue, Shinshu University. Kaempferol 7-*O*-glucoside and quercetin 7-*O*-glucoside were prepared by bioconversion in our laboratory as previously reported (Dorjjugder and Taguchi 2022). Other reagents were obtained from Sigma-Aldrich, Nacalai Tesque, Tokyo Chemical Industry, Kanto Chemicals (Tokyo, Japan) or FUJIFILM Wako Pure Chemical.

### Isolation and heterologous expression of candidate genes

Total RNA extraction from cultured sweet potato cells (a kind gift from Dr. Masayuki Nozue, Shinshu University), first-strand cDNA synthesis, and PCR using a primer constructed from the consensus sequence of UGT (UGT-F1, Supplementary Table S1) were performed as previously described (Taguchi et al. 2003). The fragments were cloned into the pT7Blue-T vector (Merck Millipore, Darmstadt, Germany) using TA cloning. After confirming the DNA sequences, candidate gene fragments showing homology to UGT84 and UGT74 were selected and their full-length cDNAs were isolated by 5′-RACE reaction using GeneRacer Kit (Thermo Fisher Scientific, Tokyo, Japan). The full-length cDNA of the candidate genes was amplified from the cDNA using specific primers (Supplementary Table S1) and Phusion DNA polymerase (New England Biolabs, MA, USA) and cloned into the pCold I vector (Takara Bio, Kusatsu, Japan). The plasmid was introduced into *E. coli* BL 21(DE3) (Merck Millipore), and the recombinant enzymes were expressed according to the manufacturer’s instructions (Takara Bio). The recombinant enzyme was extracted and purified using a nickel affinity column (His GraviTrap, Cytiva, Tokyo, Japan) (Supplementary Figure S1) and concentrated using Amicon-Ultra-15 Ultracel-30K (Merck Millipore), and then added with 1 mM UDP-glucose to stabilize enzyme activity.

### Enzyme assay

The reaction mixture (50 µl) was composed of the purified IbGT1 (0.4 µg) in 100 mM potassium phosphate buffer (pH 8.0) or 100 mM sodium acetate buffer (pH 5.0), 5 mM 2-mercaptoethanol, 0.01% bovine serum albumin, and 1 mM UDP-glucose. The reaction was initiated with the addition of cinnamates (200 µM) or flavonoids (20 µM) at 30°C for 10–30 min. Then the reaction was stopped by adding 5 µl of 1 M HCl. After the addition of 100 µl of methanol and 5 µl of internal standard (1 mM chrysin), the reaction mixture was centrifuged (18,800×g, 4°C for 10 min) and the supernatant was filtrated for ultra-performance liquid chromatography-mass spectrometry (UPLC-MS) analysis. The reaction for pH preference was performed in the 60 mM sodium acetate buffer (pH 3.5–5.5) or potassium phosphate buffer (pH 5.5–9.0). To determine the kinetic parameters of *trans*-cinnamic acid and *p*-coumaric acid with IbGT1, their concentrations were varied from 50 to 3,000 µM and 50 to 2,000 µM, respectively, at a fixed UDP-glucose concentration of 1 mM. To determine the kinetic parameters of UDP-glucose with IbGT1, its concentration was varied from 10 µM to 2,000 µM at a fixed *p*-coumaric acid concentration of 2 mM. Kinetic parameters were obtained by fitting the kinetics data to the Michaelis–Menten equation using Hyper 32 software.

### UPLC-MS analysis

UPLC-MS analysis was performed using an ACQUITY UPLC SQD system (Waters, Milford, MA, USA) equipped with an octadecylsilyl (ODS) column (2.1×50 mm, ACQUITY UPLC BEH C18 Column, Waters), a diode array detector and an electron spray ionization probe (negative mode), as described previously (a gradient of 20% to 60%, LC-method A, Dorjjugder and Taguchi 2022). For the analysis of hydroxycinnamates and benzoates, the column was eluted with 5% solvent B (methanol supplemented with 0.1% formic acid) in solvent A (0.1% formic acid) for 0.5 min, followed by a gradient from 5% to 65% solvent B in A for 3 min, 65% solvent B in A for 1.5 min, and finally 20% solvent B in A for 2 min at a flow rate of 0.25 ml min^−1^ and temperature of 40°C (LC-method B). The retention time of MS peaks were delayed by about 0.08 min over that of the diode array.

### Substrate bioconversion

Bioconversion was performed using methods described previously (Dorjjugder et al. 2021). In short, *E. coli* cells expressing the recombinant IbGT1 enzyme (Ec-IbGT1) was resuspended in M9 medium containing 2% glucose at a cell density was OD_600_=2.5–3.5. Glucose was added to provide the glucose moiety of UDP-glucose and as an energy source for UDP-glucose production (Ito et al. 2014). The Ec-IbGT1 (3 ml) was supplemented with 200 µM each of cinnamates or benzoates, or 50 µM each of quercetin and kaempferol. The bioconversion process was performed at 30°C with 150 rpm for 3–6 h. For the scaled-up bioconversion, the Ec-IbGT1 (360 ml) was repeatedly administered 200 µM of sinapic acid, *trans*-cinnamic acid or *p*-hydroxybenzoic acid as dimethyl sulfoxide (DMSO) solution. The resulting acyl-glucoses were purified from the culture medium using an ODS column in the same method as described (Dorjjugder et al. 2021).

### NMR analysis

NMR spectra were recorded using a Bruker Avance Neo 400 spectrometer (Bruker BioSpin, Yokohama, Japan) with DMSO-*d_6_* as a solvent, and were compared with referenced NMR spectra of related compounds (Chen et al. 2008; Miyake et al. 2007).

## Results and discussion

We attempted to isolate the cDNAs of cinnamate glucosyltransferases from sweet potatoes using homology-based PCR. The first PCR was performed against the cDNA of sweet potato cells using primers that were constructed from the consensus sequences of UGTs (Taguchi et al. 2003) and oligo-dT. Among 27 UGT-like fragments obtained, five clones showing homology with known major carboxylic acid UGTs (Group L or orthologous group 14, UGT84, 75 and 74, Lim et al. 2001, 2002; Yonekura-Sakakibara and Hanada 2011) were selected and their full-length cDNAs were isolated by 5′-RACE reaction and designated *IbGT1-5* (Supplementary Table S2). The full-length cDNAs of the candidate genes were cloned into the pCold I vector and heterologously expressed in *E. coli*.

**Figure 1:**
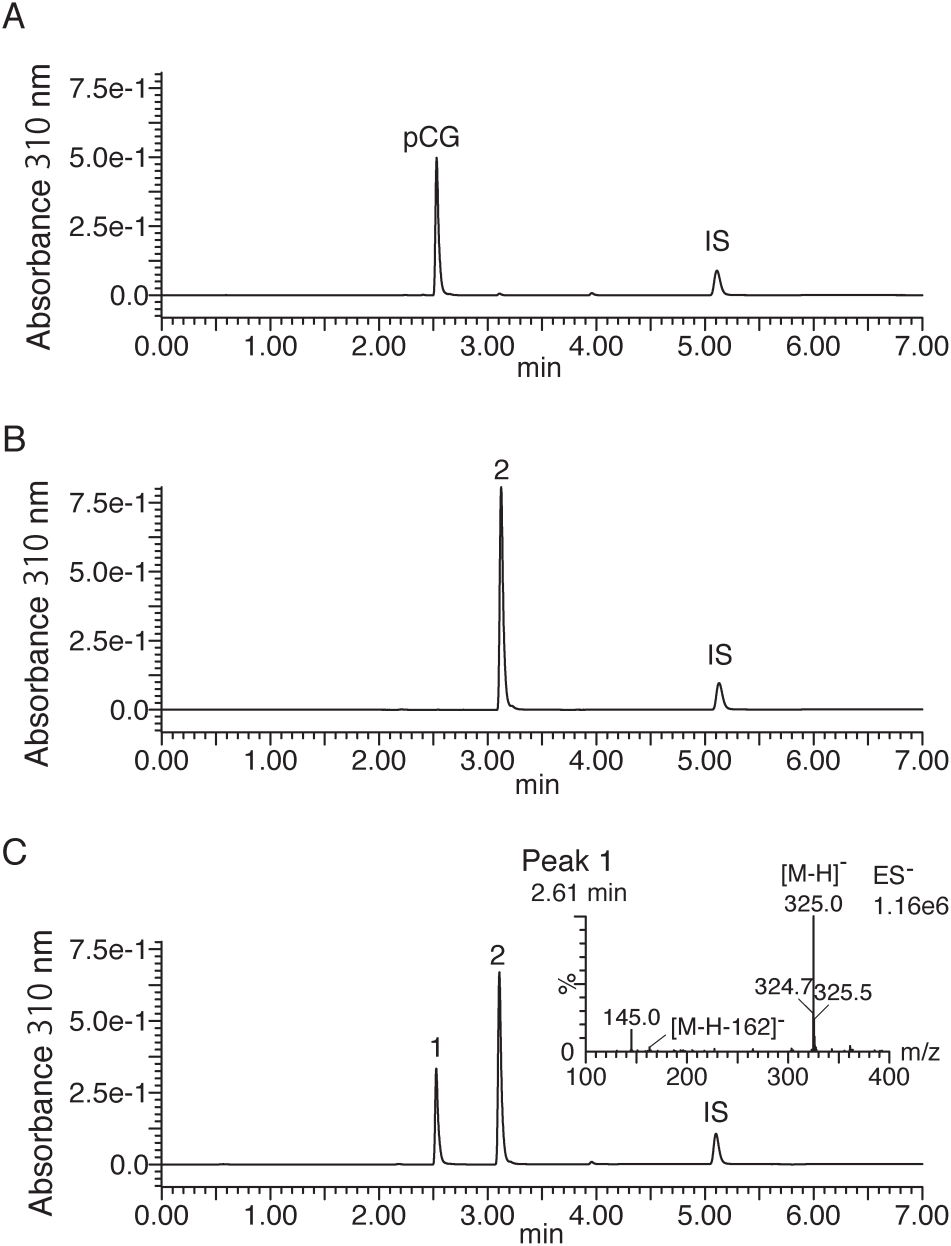
Figure 1. UPLC-MS analysis of the recombinant IbGT1 reaction against *p*-coumaric acid. Each panels show the standard compound of *p*-coumaroyl glucose (A), and the reaction products at 0 min (B) and 10 min (C) separated by LC-method B. The MS spectrum corresponding to the product is shown in the panel. The product displays [M-H]^−^ ion at *m*/*z* 325 and [M-H-162]^−^ ion at *m*/*z* 163. Peak identification: pCG and 1, *p*-coumaroyl glucose; 2, *p*-coumaric acid; IS, internal standard (chrysin).

The recombinant enzymes were evaluated for enzymatic activity using *p*-coumaric acid and *trans*-cinnamic acid (the structures of the substrates are shown in Supplementary Figure S2). Of these, recombinant IbGT1 (DDBJ accession No. AB909370, named UGT84A20 by the UGT nomenclature committee) reacted with both compounds (Figure 1, Supplementary Figure S3). The reaction product of IbGT1 with *p*-coumaric acid was confirmed as *p*-coumaroyl glucose by comparison with the authentic compound (Figure 1, Table 1), proving that IbGT1 is a carboxylic acid glucosyltransferase. The reaction product with *trans*-cinnamic acid is considered to be *trans*-cinnamoyl glucose, as it has only one functional group that can be glucosylated by UGT, which was later confirmed (Supplementary Figure S3, Table 1). In contrast, recombinant IbGT3 (UGT74AA1, DDBJ accession No. AB909372) reacted weakly with *trans*-cinnamic acid (0.56 nkat mg_protein^−1^ at pH 5.0), and the others showed no activity with these compounds. Therefore, further experiments were conducted using IbGT1. The maximum glucose ester-forming reaction of recombinant IbGT1 with *p*-coumaric acid and *trans*-cinnamic acid was observed at pH 5.0–5.5 (Supplementary Figure S4), which was similar to reported cinnamate glucosyltransferases (Hall and De Luca 2007; Ono et al. 2016). The kinetic parameters of IbGT1 enzyme with these compounds and UDP-glucose are listed in Table 2. These parameters were comparable to or better than those reported for UGT84 enzymes (Supplementary Table S3), suggesting that IbGT1 is highly active toward cinnamate substrates.

**Table 1:** Table 1. Substrate specificity of the recombinant IbGT1.

Substrate	Activity (nkat mg_protein^−1^)	
	Sodium acetate buffer (pH 5.0)	Potassium phosphate buffer (pH 8.0)
*p*-coumaric acid	22.6±0.80	8.92±0.75
*trans*-cinnamic acid	9.25±0.27	3.79±0.05
*p*-methoxycinnamic acid	17.8±0.73	6.83±0.11
*o*-coumaric acid	16.5±0.59	6.80±0.06
caffeic acid	21.5±0.17	8.44±0.18
ferulic acid	24.4±1.13	9.19±0.50
sinapic acid	23.3±0.08	8.40±0.24
benzoic acid	4.45±0.09	2.30±0.08
*o*-hydroxybenzoic acid	N.D.	N.D.
*p*-hydroxybenzoic acid	12.2±0.88	6.52±0.12
kaempferol	N.D.	4.77±0.11^a^
quercetin	N.D.	3.40±0.07

N.D., no significant peaks of glucosylated products were detected. ^a^ Sum of two products (7-*O*-glucoside and 3-*O*-glucoside). Average±S.D. (*n*=3).

**Table 2:** Table 2. Kinetic parameters of the recombinant IbGT1.

Substrate	*K*_m_ (µM)	*k*_cat_ (s^−1^)	*k*_cat_/*K*_m_ (µM^−1^ s^−1^)
*p*-coumaric acid	251±32.6	7.72±0.38	30.7
*trans*-cinnamic acid	232±40.2	1.85±0.12	7.96
UDP-glucose ^a^	76.5±10.9	5.42±0.24	71.3

^a^ *p*-coumaric acid was used as a sugar acceptor.

The substrate specificity of IbGT1 was further studied. The enzymes were incubated with several phenolics as glucosyl acceptors (Supplementary Figure S2), using UDP-glucose as a sugar donor (Table 1). In a preliminary experiment at pH 8.0 (a common reaction pH for UGTs), IbGT1 also showed glucosylation activity toward quercetin. When the pH was varied between 3.5–9.0, the optimum pH of the reaction with quercetin was 8.0–9.0, and few products were observed under acidic conditions (Supplementary Figure S4). Therefore, the reaction was performed at pH 8.0 in addition to at pH 5.0, an optimum pH for cinnamates (Supplementary Figure S4). IbGT1 exhibited significant glucosylating activity toward all tested cinnamates at pH 5.0, but the activity decreased by approximately half at pH 8.0. Its activity was strong toward ferulic acid, sinapic acid, *p*-coumaric acid, and caffeic acid; however, it was weaker toward *trans*-cinnamic acid (Table 1). This result was slightly different from that of a partially purified enzyme from sweet potato, which showed strong activity with *trans*-cinnamic acid and weak activity with sinapic acid (Shimizu and Kojima 1984). The partially purified enzyme might have contained several isozymes, which may have resulted this difference.

IbGT1 also showed significant activity against flavonols at pH 8.0 but not at pH 5.0 (Table 1), which is probably because the hydroxy groups of flavonoids are difficult to dissociate under acidic conditions, given their pKa. When quercetin and kaempferol were used as substrates, the enzyme produced their 7-*O*-glucoside preferentially with several minor products (Supplementary Figure S5). These results show that the enzyme can react with both carboxyl and hydroxyl groups. It has been reported that some UGT84 enzymes can glycosylate flavonoids in addition to cinnamates (Hall and De Luca 2007), and that some UGT84 enzymes catalyze *C*-glycosylation of flavonoids (Mashima et al. 2019; Sasaki et al 2015). This may reflect the evolutionary history of the UGT84 enzymes.

IbGT1 is also active with *p*-hydroxybenzoic acid and benzoic acid, but not with *o*-hydroxybenzoic acid (salicylic acid), similar to some UGT84 enzymes, such as UGT84A1 (Lim et al. 2001, 2002). It should be noted that the activity of recombinant IbGT1 was at a higher level for both of cinnamates and benzoates compared to those of other UGT84 enzymes. For example, the activity of IbGT1 against sinapic acid and *p*-hydroxybenzoic acid were 23.3 and 12.2 nkat mg_protein^−1^, respectively. The activity of three UGT84s, FaGT2, VLRSgt, and UGT84A23 against sinapic acid and *p*-hydroxybenzoic acid were 1.2–4.3 and 0.7–2.3 nkat mg_protein^−1^, respectively (Hall and De Luca 2007; Lunkenbein et al. 2006; Ono et al. 2016). UGT84A2 and UGT84A3 from *Arabidopsis* showed relatively high activity toward sinapic acid (13.4 and 11.8 nkat mg_protein^−1^, respectively), but do not show the activity against *p*-hydroxybenzoic acid (Lim et al. 2001, 2002). These results suggested the high potential of IbGT1 as a biocatalyst.

**Figure 2:**
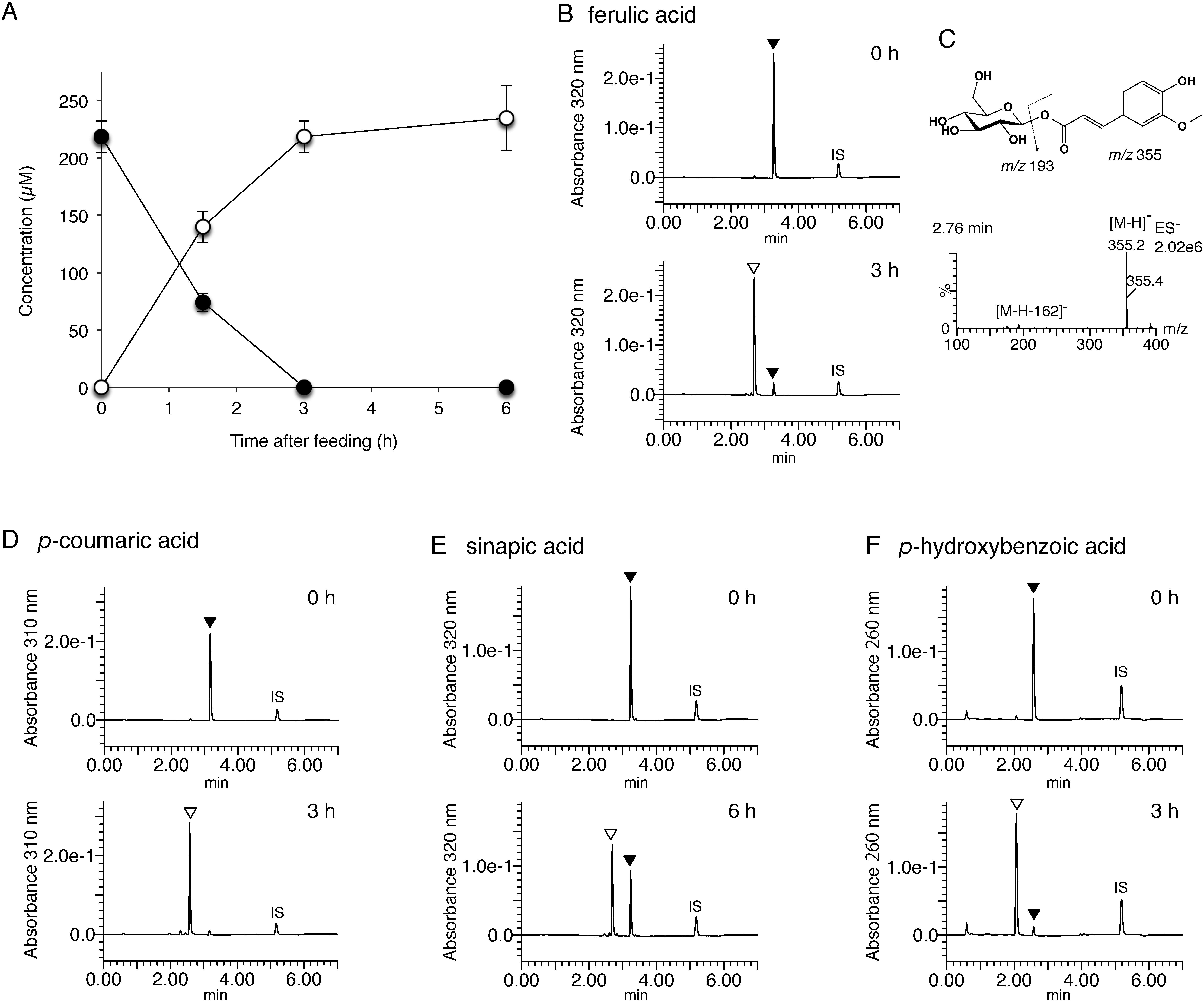
Figure 2. Bioconversion of cinnamates and benzoates to their glucosides by the Ec-IbGT1 system. A: Time course of ferulic acid (closed circle) to its glucoside (open circle); B: UPLC profiles of the culture media at 0 and 3 h after the addition of ferulic acid; C: MS spectrum of the product in B. The product displays [M-H]^−^ ion at *m*/*z* 355 and [M-H-162]^−^ ion at *m*/*z* 193; D–F: Conversion results of *p*-coumaric acid (D), sinapic acid (E) and *p*-hydroxybenzoic acid (F) at 0 h and 3 or 6 h. LC-method B was used for the separation. Peak identification: open triangles, product; closed triangles, substrate; IS, internal standard (chrysin).

As IbGT1 exhibited remarkable glucosylation activity against cinnamates and benzoates, bioconversion was performed using *E. coli* cells expressing the recombinant IbGT1 enzyme (Ec-IbGT1) for its potential as a whole-cell biocatalyst. The Ec-IbGT1 (3 ml) was supplemented with 200 µM each of cinnamates or benzoates, cultured at 30°C with 150 rpm for 3–6 h. The bioconversion of ferulic acid by Ec-IbGT1 is shown in Figure 2A–C. The administered ferulic acid mostly disappeared from the culture medium in 3 h and a metabolite accumulated instead (Figure 2A, B). The metabolite displayed [M-H]^−^ ion at *m*/*z* 355 and [M-H-162]^−^ ion at *m*/*z* 193, which was corresponded to feruloyl glucose (Figure 2C). Similarly, the Ec-IbGT1 converted *trans*-cinnamic acid, *p*-coumaric acid, *o*-coumaric acid, and *p*-hydroxybenzoic acid into their glucose esters within 3 h, in contrast, conversions of caffeic acid and sinapic acid were not completed in 6 h (Figure 2D–F, Supplementary Figure S6). Similar to other glycosides produced by bioconversion in *E. coli*, almost all of the products were released into the culture media. Interestingly, the efficiency of this bioconversion did not necessarily correspond to the activity of IbGT1 toward each substrate. For example, *trans*-cinnamic acid and *p*-hydroxybenzoic acid were fewer active substrates in the enzyme assay but were more efficiently converted in the Ec-IbGT1 system than sinapic acid and ferulic acid, which are more active substrates in the enzyme assay. Unlike the enzyme reaction *in vitro*, where the enzyme can access the substrates freely, whole-cell biocatalyst requires the substrates to be incorporated into the cells across the cell membrane, which may affect the efficiency of the reaction. We also examined for the biotransformation of flavonols (50 µM each) into their glucosides using Ec-IbGT1. Quercetin and kaempferol were converted into their 7-*O*-glucosides but was not completed in 6 h, and the products were a mixture of several glucosides (Supplementary Figure S6). The conversion rate was much lower than that of Ec-NtGT2, which can produce 7-*O*-glucosides of flavonols as the sole product within 1 h under the same conditions (Dorjjugder and Taguchi 2022), so that this system is not suitable for the production of flavonoid glucosides.

Scaled-up bioconversion was examined using sinapic acid as a model. Sinapic acid is a representative cinnamate distributed in various plants and has antioxidant properties (Kylli et al. 2008), but it is only slightly soluble in water, which could be improved through glycosylation. The Ec-IbGT1 was repeatedly supplemented with sinapic acid (200 µM) at 0 h, 4.5 h, and 8.5 h to aim to scale up a final amount of the product. The introduction of a total of 51.5 mg sinapic acid into 360 ml of the cell culture resulted in a yield of approximately 69 mg (191 mg l^−1^) of sinapoyl glucose, with a bioconversion rate about 78% after 30 h-incubation (Supplementary Figure S7A). The resulting sinapoyl glucose was purified from the culture medium and analyzed by NMR (Supplementary Table S4). The obtained signals corresponded to those of standard sinapoyl glucose (Miyake et al. 2007). Moreover, correlations between H-1′(glucose moiety, δ 5.48) and C-9 (165.8) were observed in heteronuclear multiple bond correlation (HMBC) analysis (Supplementary Figure S8A), confirming that the product is sinapoyl glucose. Bioconversion of *trans*-cinnamic acid into *trans*-cinnamoyl glucose was also tested as in the same manner, with the administration of a total of 53 mg *trans*-cinnamic acid in 360 ml of the cell culture resulted in a yield of approximately 83 mg (231 mg l^−1^) of *trans*-cinnamoyl glucose, with a bioconversion rate about 74% after 30 h-incubation (Supplementary Figure S7B). The NMR spectrum and results of the HMBC analysis also showed that the product was *trans*-cinnamoyl glucose (Supplementary Figure S8B, Supplementary Table S4). When the incubation time for the bioconversion of cinnamates was prolonged, several minor byproducts were observed (Supplementary Figure S7A, B). They showed mostly the same MS spectrum as the main product (glucose ester), so it is possible that they are transacylation products of the main product, such as 6-*O*-acylated glucose, but could not be determined due to their low amounts.

Subsequently, *p*-hydroxybenzoic acid was tested as a substrate for the scaled-up bioconversion process. Four cycles of *p*-hydroxybenzoic acid administration (200 µM) at every 3 h, with the addition of a total of 40 mg *p*-hydroxybenzoic acid in 360 ml of the cell culture resulted in a yield of approximately 82 mg (228 mg l^−1^) of *p*-hydroxybenzoyl glucose, with a bioconversion rate reached 95% after 20 h-incubation (Supplementary Figure S7C). The product peak corresponded to that of the authentic compound, and the NMR signal (Supplementary Figure S9, Supplementary Table S4) corresponded to those of the reported *p*-hydroxybenzoyl glucose (Chen et al. 2008), proving that the product was *p*-hydroxybenzoyl glucose.

**Figure 3:**
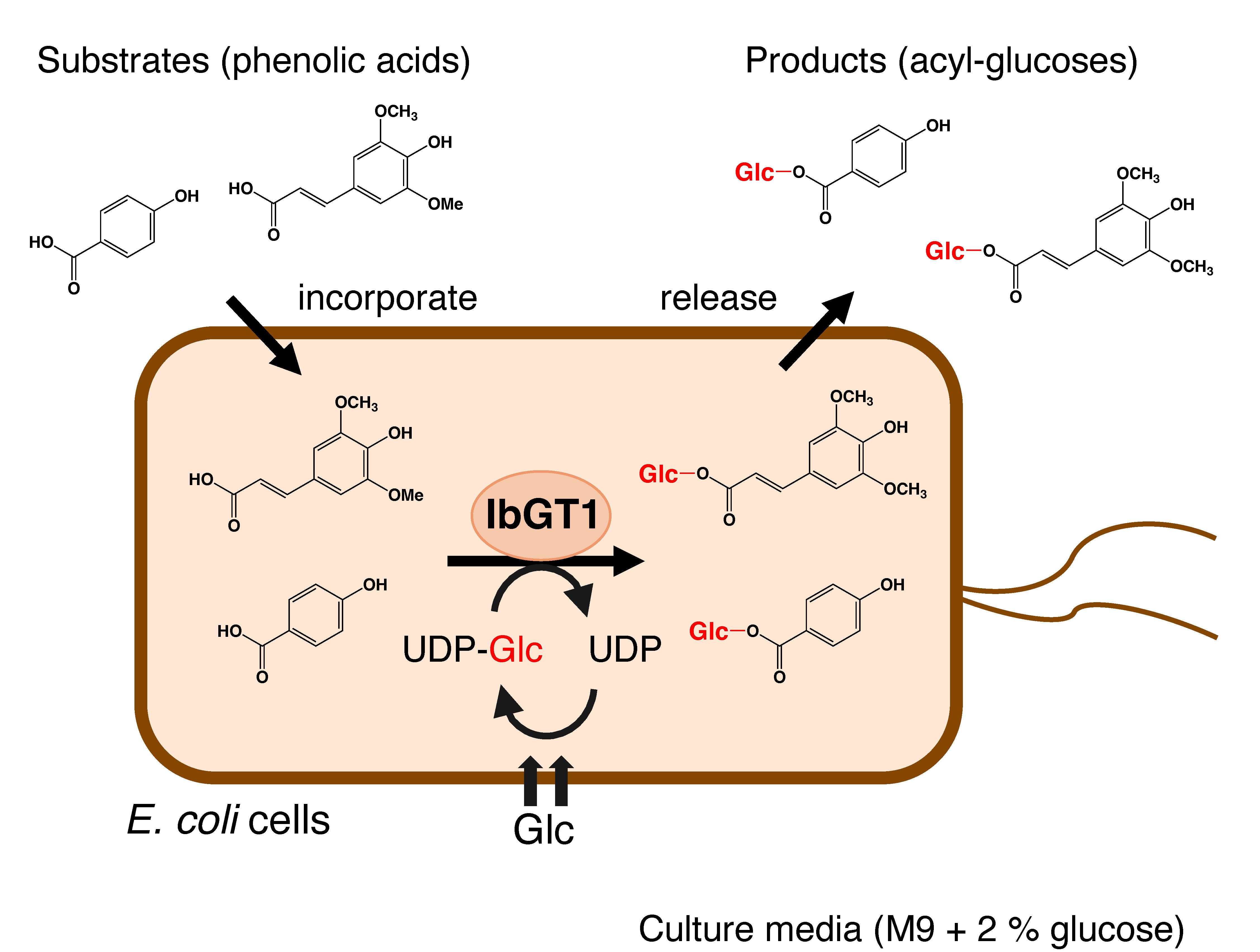
Figure 3. A schematic diagram for bioconversion of phenolic acid into their glucose ester by *E. coli* expressing IbGT1 (Ec-IbGT1). The administered phenolic acids were incorporated into the cells, converted to their glucose esters by IbGT1, and then released into the culture medium. Glc: glucose.

Han et al. (2016) reported production of feruloyl glucoside using *E. coli* cells expressing a regioselective glucosyltransferase AtUGT71C1 from *Arabidopsis*; however, the maximum yield was 1.8 µM (0.6 mg l^−1^) in 1 h. The enzymatic production of glucose esters from cinnamates and benzoates has been reported using a recombinant sinapate glucosyltransferase (GgSGT) enzyme coupled with the recycling of UDP-glucose driven by sucrose synthase. This produced 2.4–7 mg of the products in a 30 ml reaction at the laboratory scale (Matsuba et al. 2008). Recently, enzymatic glucose ester synthesis via transglycosylation using Os9BGlu31 and *p*-nitrophenyl glucose was reported. This system produced approximately 10 mg of the products in a 100 ml reaction at the laboratory scale (Suyanto et al. 2024). Ec-IbGT1 system converted administered cinnamates and benzoates into their glucose esters with conversion rates of 70%–95% in 2–6 h and could be further scaled up more than 100 mg l^−1^ with repeated administration of the substrates. A schematic of the Ec-IbGT1 system is shown in Figure 3. These results clearly suggest the practical usefulness of the Ec-IbGT1 system for the simple and efficient bioproduction of acyl-glucoses in various fields.

In this study, we isolated and characterized IbGT1 (UGT84A20) from sweet potato, which is highly active toward both cinnamates and benzoates, and constructed a simple and efficient bioconversion system. It is important to identify the enzymes that are suitable for each reaction to obtain better conversion efficiency.

## Abbreviations

DMSO: dimethyl sulfoxide
5′-RACE: rapid amplification of cDNA ends
HMBC: heteronuclear multiple bond correlation
ODS: octadecylsilyl
UDP-glucose: uridine 5′-diphosphoglucose
UGT: uridine 5′-diphosphate-sugar dependent glycosyltransferase
UPLC-MS: ultra-performance liquid chromatography-mass spectrometry

## Description of Supplementary Files

Supplementary Table S1 Primers used in this study.

Supplementary Table S2 List of UGTs isolated from *Ipomoea batatas* in this work.

Supplementary Table S3 Comparison of the kinetic parameters of UGT84 enzymes.

Supplementary Table S4 NMR assignment of the purified compounds produced by bioconversion.

Supplementary Figure S1 SDS-PAGE analysis of the recombinant IbGT1 expressed in *E. coli*.

Supplementary Figure S2 Chemical structures of substrates used for the enzyme assay of IbGT1.

Supplementary Figure S3 UPLC-MS analysis of the recombinant IbGT1 reaction against *trans*-cinnamic acid.

Supplementary Figure S4 pH preference of the recombinant IbGT1 reaction.

Supplementary Figure S5 UPLC analysis of the recombinant IbGT1 reaction against quercetin and kaempferol.

Supplementary Figure S6 Bioconversion of cinnamates and flavonols by the Ec-IbGT1 system.

Supplementary Figure S7 Results of the scaled-up bioconversion by Ec-IbGT1 after repeated addition of the substrates.

Supplementary Figure S8 Heteronuclear multiple bond correlation (HMBC) analysis of the compounds produced by bioconversion using Ec-IbGT1 system.

Supplementary Figure S9 NMR analysis of *p*-hydroxybenzoyl glucose produced by bioconversion using Ec-IbGT1.
