# Supplementary material for: Production of cinnamates and benzoates glucose esters by bioconversion using Escherichia coli expressing a glucosyltransferase from sweet potato
Source: Plant Biotechnol (Tokyo). 2024 Sep 25;41(3):295–302. doi: 10.5511/plantbiotechnology.24.0705a (PMC11921131; doi:10.5511/plantbiotechnology.24.0705a)
Supplement: Supplementary Data [file plantbiotechnology-41-3-24.0705a-s001.pdf]

Supplementary Files

for

Production of cinnamates and benzoates glucose esters by bioconversion using *Escherichia coli* expressing a glucosyltransferase from sweet potato

Yuki Kobayashi[†], Nasanjargal Dorjjugder[†], and Goro Taguchi[*]

Department of Applied Biology, Faculty of Textile Science and Technology, Shinshu University, 3-15-1 Tokida, Ueda 386-8567, Japan.

[*]Corresponding author. e-mail : gtagtag@shinshu-u.ac.jp
[†]These authors contributed equally to this work.

Supplementary Table S1 Primers used in this study.

Name	Primer sequence (5′→3′)
dT-T3	ATTAACCCTCACTAAAGGGTTTTTTTTTTTTTTTTVV
T3-promoter	AATTAACCCTCACTAAAGGG
pCold-F	ACGCCATATCGCCGAAAGG
pCold-R	GGCAGGGATCTTAGATTCTG
UDP-F1	GCTCTAGACKCATTGYGGNTGGAAYTC
IbGT1-exF	GGTCTAGACATATGGGGTCTGAAAACGGCGGCGTTCCACC
IbGT1-ex R	GGGCTCGAGTTAGTGTTTTCCGCCACCGCATTTG
IbGT2-ex F	GGTCTAGACATATGAACAAGCACCATCATT
IbGT2-ex R	GGCTCGAGACCTGTGTTCTCTAATGGAAA
IbGT3-ex F	GGTCTAGACATATGGAGAACCATGAAAACAAGG
IbGT3-ex R	GGCTCGAGATTAGTCTGTCATGAATTTGG
IbGT4-ex F	GGTCTAGACATATGGCCGAAATGTCTAC
IbGT4-ex R	GGGCTCGAGTTACATAGAAATAGCAGACAC
IbGT5-ex F	GGTCTAGACATATGGAGAGAGAAAAGAGAGCAC
IbGT5-ex R	GGGCTCGAGATCAAACATGGGATAGTGATGC
IbGT1 RACE1	GACAGCAACGTGGCGCCGGTGTTA
IbGT1 RACE2	TTCTCCACCTCGTCCCGGGTAAT
IbGT2 RACE1	CCCTCTCTCCCCTCCTCCCATTA
IbGT2 RACE2	AACAGGACTGTGGGCACGGGCGA
IbGT3 RACE1	CCTACATCCACCGCCTCTCTGCAA
IbGT3 RACE2	TCTGCGCCCTCACCCCAGTTTTC
IbGT4 RACE1	GTCCGGCTCACTGATTGGCGTTA
IbGT4 RACE2	CCCTACATCCCAAACATCGACGAGAT
IbGT5 RACE1	CACTCACTGCCTCCTTAGCCAAATG
IbGT5 RACE2	CCTCCCCCAAAACCTCCCTAACAC

Supplementary Table S2 List of UGTs isolated from *Ipomoea batatas* in this work.

Gene Name	UGT name	Length (aa)	Molecular weight	pI	Accession No.
IbGT1	UGT84A20	478	52,845	5.4	AB909370
IbGT2	UGT75S1	463	51,453	5.8	AB909371
IbGT3	UGT74AA1	460	52,626	4.9	AB909372
IbGT4	UGT74AB1	450	49,572	5.1	AB909373
IbGT5	UGT74T3	459	50,980	5.7	AB909374

Supplementary Table S3 Comparison of the kinetic parameters of UGT84 enzymes.

Enzyme	Source	Substrate	K_m (mM)	V_{max} (nkat mg^{-1})	k_{cat} (s^{-1})	k_{cat}/K_m (mM^{-1} s^{-1})	Reference
IbGT1 (UGT84A20)	sweet potato	*p*-coumaric acid	0.251±0.033		7.72±0.38	30.7	This study
	(*Ipomoea batatas*)	*trans*-cinnamic acid	0.232±0.040		1.85±0.12	7.96	
UGT84A1	mouse-ear cress	*p*-coumaric acid	0.4	13.07			Lim et al (2001)
	(*Arabidopsis thaliana*)	*trans*-cinnamic acid	0.71	4.8			
UGT84A3	mouse-ear cress	*p*-coumaric acid	0.18	7.7			Lim et al (2001)
	(*Arabidopsis thaliana*)	*trans*-cinnamic acid	0.59	16.95			
		ferulic acid	0.24	15.08			
FaGT2	strawberry	*trans*-cinnamic acid	0.357±0.082	2.34±0.53		0.42	Lunkenbein (2006)
	(*Fragaria × ananassa*)	*p*-coumaric acid	0.604±0.121	2.69±0.54		0.29	
VLRSgt	grape (*Vitis labrusca*)	sinapic acid	27±0.40		4.68±0.61	0.173	Hall and De Luca (2007)
UGT84A23	pomegranate	garic acid	0.89 ± 0.07		0.52 ± 0.03	0.58	Ono et al (2016)
	(*Punica granatum*)	*p*-coumaric acid	0.94 ± 0.13		0.52 ± 0.07	0.55	
UGT84A77	Chinese olive (*Canarium album*)	garic acid	0.109±0.0211		3.71±0.18	34.1	Ye et al (2021)

Supplementary Table S4 NMR assignment of the compounds produced by scaled-up bioconversion.

Proton and carbon NMR spectra				
sinapoyl glucose				
aglycon				
	^{1}H (δ)	9.01 (1H, *s*, Ar-OH)	^{13}C (δ)	165.8 (C-9)
		7.65 (1H, *d*, *J*=15.8 Hz, H-7)		148.4 (C-3 and C-5)
		7.06 (2H, *s*, H-2 and H-6)		147.0 (C-7)
		6.55 (1H, *d*, *J*=15.9 Hz, H-8)		139.0 (C-4)
		3.81 (6H, *s*, methoxy)		124.6 (C-1)
				114.7 (C-8)
				106.8 (C-2 and C-6)
				56.5 (methoxy)
sugar moiety				
	^{1}H (δ)	5.48 (1H, *d*, *J*=8.0 Hz, H-1′)	^{13}C (δ)	94.6 (C-1′)
		5.28 (1H, sugar-OH)		78.2 (C-3′)
		5.12 (1H, sugar-OH)		76.9 (C-5′)
		5.02 (1H, sugar-OH)		72.9 (C-2′)
		4.59 (1H, sugar-OH)		69.9 (C-4′)
		3.68 (1H, *d*, *J*=11.4 Hz, H-6′α)		61.0 (C-6′)
		3.47 (1H, *m*, H-6′β)		
		3.30–3.12 (4H, *m*, H-2′–H-5′)		
trans-cinnamoyl glucose				
aglycon				
	^{1}H (δ)	7.81 (1H, *d*, *J*=15.9 Hz, H-7)	^{13}C (δ)	165.5(C-9)
		7.80 (2H, *dd*, *J*=7.4 Hz, 2.0 Hz, H-2 and H-6)		146.3 (C-7)
		7.51(2H, *d*, *J*=2.3 Hz, H-3 and H-5)		134.3 (C-1)
		7.49 (1H, *m*, H-4)		131.2 (C-4)
		6.71 (1H, *d*, *J*=16.1 Hz, H-8)		129.5 (C-3 and C-5)
				129.0 (C-2 and C-6)
				118.0 (C-8)
sugar moiety				
	^{1}H (δ)	5.55 (1H, *d*, *J*=7.9 Hz, H-1′)	^{13}C (δ)	94.9 (C-1′)
		5.39 (1H, sugar-OH)		78.4 (C-3′)
		5.20 (1H, sugar-OH)		76.9 (C-5′)
		5.10 (1H, sugar-OH)		73.0 (C-2′)
		4.59 (1H, sugar-OH)		70.0 (C-4′)
		3.73 (1H, *dd*, *J*=11.8 Hz, 3.3 Hz, H-6′α)		61.1 (C-6′)
		3.52 (1H, *dd*, *J*=12.0 Hz, 5.7 Hz, H-6′β)		
		3.36–3.19 (4H, *m*, H-2′–H-5′)		

Supplementary Table S4 continued

Proton and carbon NMR spectra				
p-hydroxybenzoyl glucose				
aglycon				
^{1}H (δ)	10.40 (1H, *s*, Ar-OH)		^{13}C (δ)	164.9 (C-7)
	7.87 (2H, *d*, *J*=8.8 Hz, H-3 and H-5)			162.8 (C-4)
	6.87 (2H, *d*, H-2 and H-6)			132.4 (C-2 and 6)
				120.2 (C-1)
				115.8 (C-3 and C-5)
sugar moiety				
^{1}H (δ)	5.53 (1H, *d*, *J*=8.0 Hz, H-1′)		^{13}C (δ)	95.0 (C-1′)
	5.35 (1H, H-4′)			78.3 (C-3′)
	5.13 (1H, H-3′)			76.9 (C-5′)
	5.04 (1H, H-2′)			73.0 (C-2′)
	4.59 (1H, H-6′)			70.0 (C-4′)
	3.66 (1H, *d*, *J*=11.7 HZ, H-6′α)			61.0 (C-6′)
	3.46 (1H, *m*, H-6′β)			
	3.30–3.12 (4H, *m*, H-2′–H-5′)			

NMR spectra were recorded as described on a Bruker Avance Neo 400 spectrometer (Bruker BioSpin, Yokohama, Japan) at 400 MHz. ^{1}H NMR (400 MHz, DMSO-d_6), ^{13}C NMR (100 MHz, DMSO- d_6).

References (for Supplementary Table S3)

Lim E-K, Li Y, Parr A, Jackson R, Ashford DA, Bowles DJ (2001) Identification of glucosyltransferase genes involved in sinapate metabolism and lignin synthesis in *Arabidopsis*. J Biol Chem 276:4344–4349

Hall D, De Luca V (2007) Mesocarp localization of a bi-functional resveratrol/hydroxycinnamic acid glucosyltransferase of Concord grape (*Vitis labrusca*). Plant J 49:579–591

Lunkenbein S, Bellido M, Aharoni A, Salentijn EM, Kaldenhoff R, Coiner HA, Muñoz-Blanco J, Schwab W (2006) Cinnamate metabolism in ripening fruit. Characterization of a UDP-glucose: cinnamate glucosyltransferase from strawberry. Plant Physiol 140:1047–1058

Ono NN, Qin X, Wilson AE, Li G, Tian L (2016) Two UGT84 family glycosyltransferases catalyze a critical reaction of hydrolyzable tannin biosynthesis in pomegranate (*Punica granatum*). PloS One 11:e0156319

Ye Q, Zhang S, Qiu N, Liu L, Wang W, Xie Q, Chang Q, Chen Q (2021) Identification and characterization of glucosyltransferase that forms 1-galloyl-*β*-D-glucogallin in *Canarium album* L., a functional fruit rich in hydrolysable tannins. Molecules 26:4650

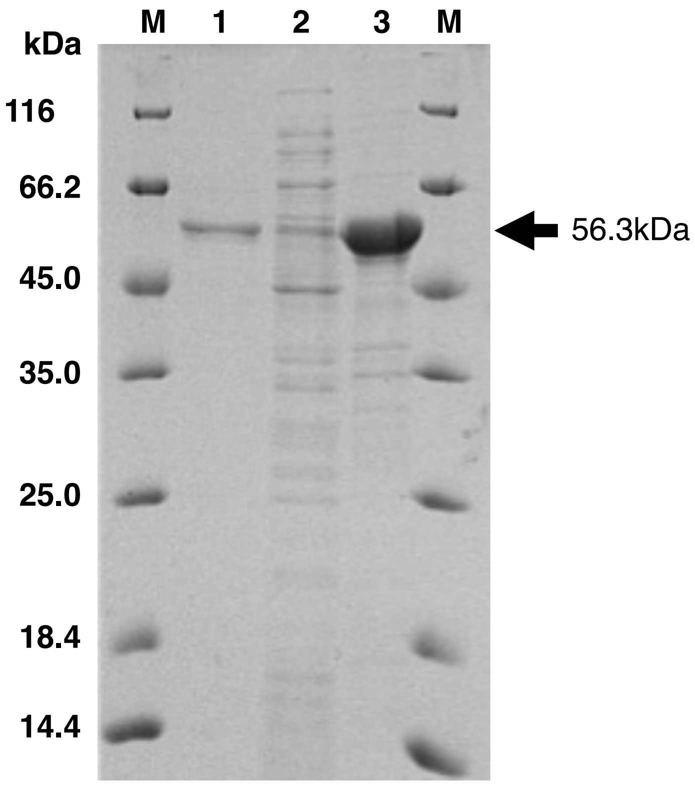

Supplementary Figure S1 SDS-PAGE analysis of the recombinant IbGT1 expressed in *E. coli*.

Proteins were separated using a 12.5 % SDS-PAGE. Lane M, Size marker (unstained protein molecular weight marker, ThermoFisher Scientific); lanes 1 and 2, insoluble (1) and soluble (2) fractions of the cell-free extract; 3, recombinant IbGT1 purified with the 6× histidine tag. Arrowheads indicate the size of recombinant IbGT1.

Cinnamates

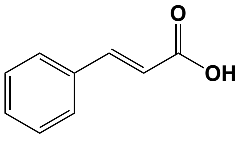

trans-cinnamic acid

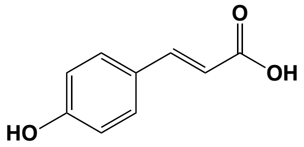

p-coumaric acid

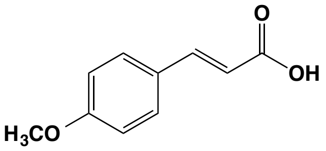

p-methoxycinnamic acid

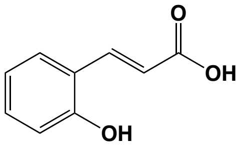

o-coumaric acid

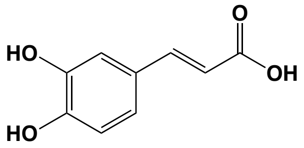

caffeic acid

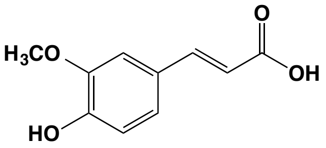

ferulic acid

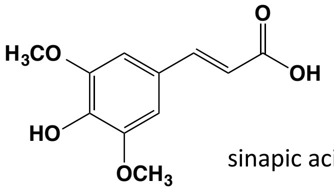

sinapic acid

Benzoates

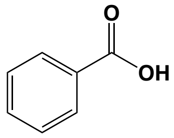

benzoic acid

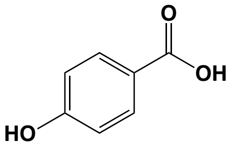

p-hydroxybenzoic acid

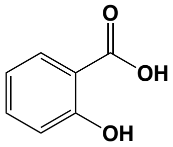

o-hydroxybenzoic acid
(salicylic acid)

Flavonols

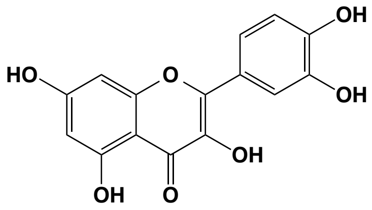

quercetin

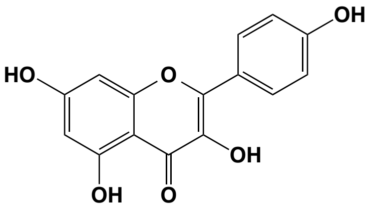

kaempferol

Supplementary Figure S2 Chemical structures of substrates used for the enzyme assay of IbGT1.

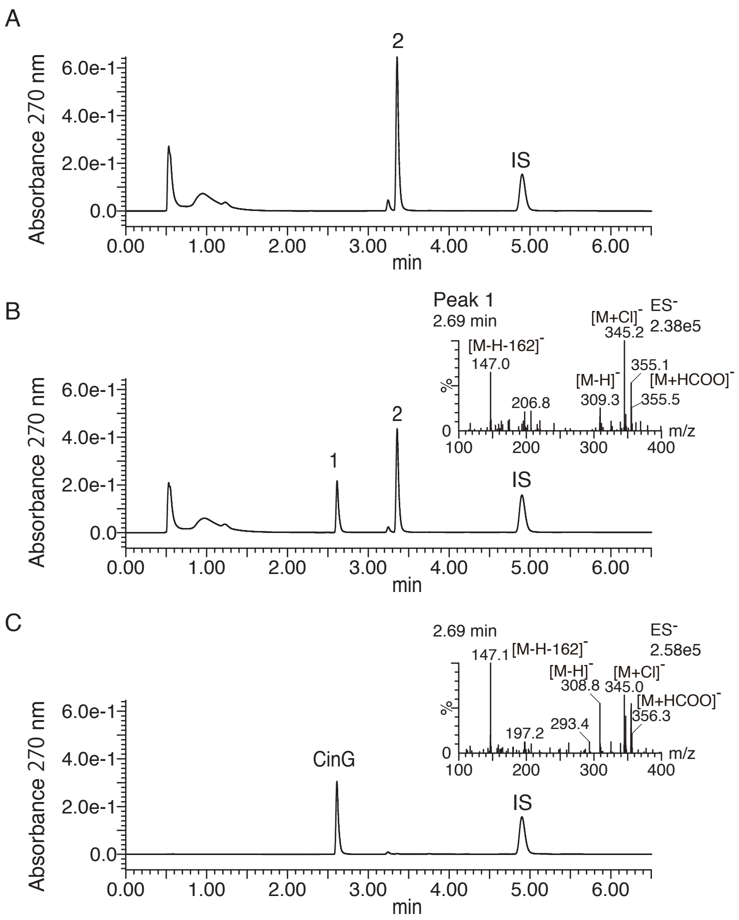

Supplementary Figure S3 UPLC-MS analysis of the recombinant IbGT1 reaction against *trans*-cinnamic acid.

The reaction products at 0 min (A) and 30 min (B), and *trans*-cinnamoyl glucose confirmed by NMR (C) were separated using LC-method A. The MS spectra corresponding to the product and standard are shown in the small panels. The product displays $[M-H]^-$ ion at *m/z* 309, $[M+Cl]^-$ ion at *m/z* 345, $[M+HCOO]^-$ ion at *m/z* 355, and $[M-H-162]^-$ ion at *m/z* 147. Peak identification: 1 and CinG, *trans*-cinnamoyl glucose; 2, *trans*-cinnamic acid; IS, internal standard (chrysin).

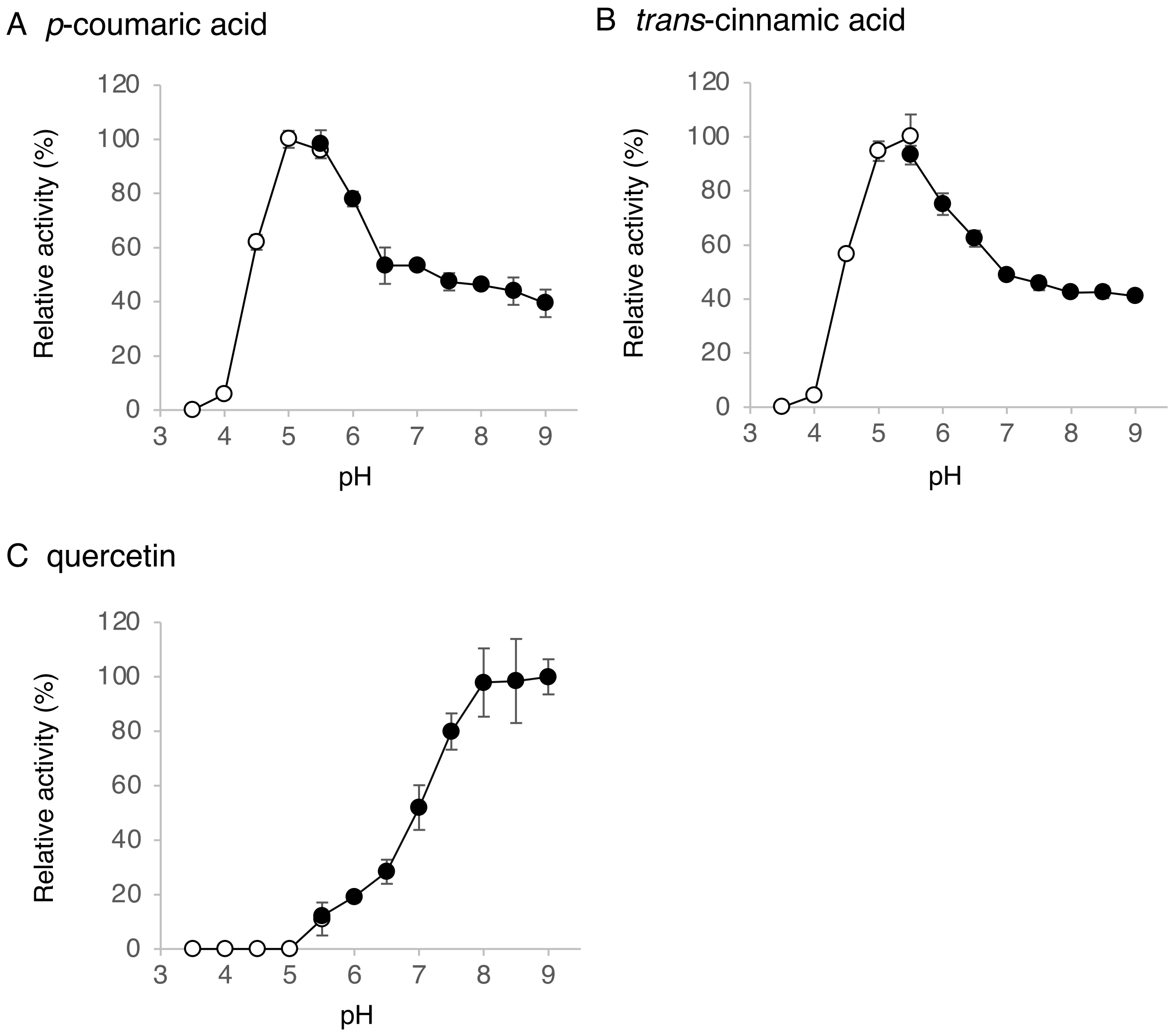

Supplementary Figure S4 pH preference of the recombinant IbGT1 reaction.

The reactions were performed using *p*-coumaric acid (A), *trans*-cinnamic acid (B), and quercetin (C) as sugar donors and UDP-glucose as a sugar acceptor, as described in the text. Values of the relative activities are average SD (n=3), with maximum activity taken to be 100 %. Open and filled circles represent the activities performed in sodium acetate buffer and potassium phosphate buffer, respectively.

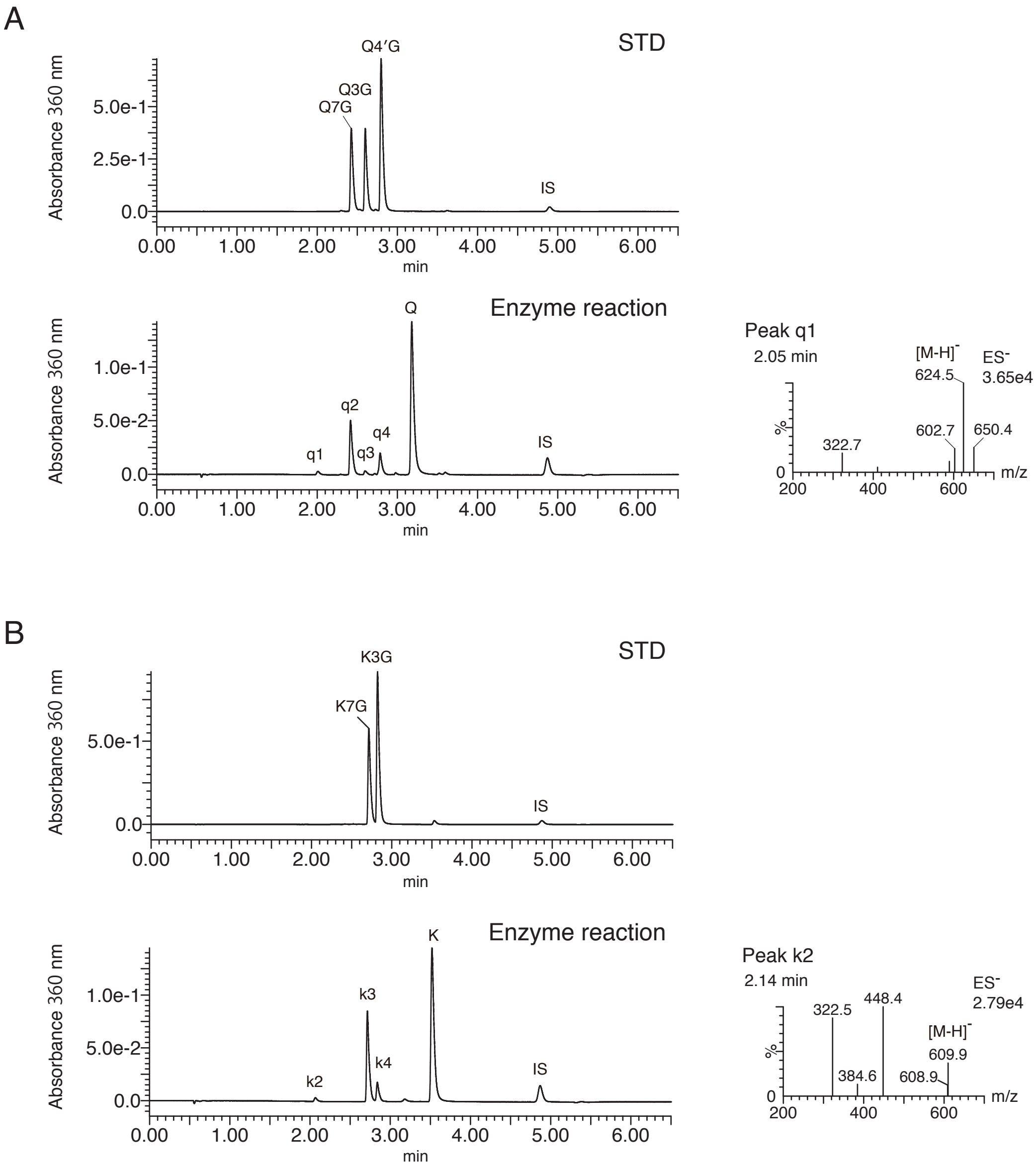

Supplementary Figure S5 UPLC analysis of the recombinant IbGT1 reaction against quercetin and kaempferol.

The reaction products of quercetin (A) and kaempferol (B) were separated using LC-method A. Upper panel: standard compounds (STD); Lower panel, enzyme reaction. The MS spectra corresponding to the products (peaks q1 and k2) were shown next to the chromatogram. Peak identification: Q, quercetin; q1, quercetin diglucoside; Q7G and q2, quercetin 7-*O*-glucoside; Q3G and q3, quercetin 3-*O*-glucoside; Q4′G and q4, quercetin 4′-*O*-glucoside; K, kaempferol; k2, kaempferol diglucoside; K7G and k3, kaempferol 7-*O*-glucoside; K3G and k4, kaempferol 3-*O*-glucoside. IS: Internal standard (chrysin).

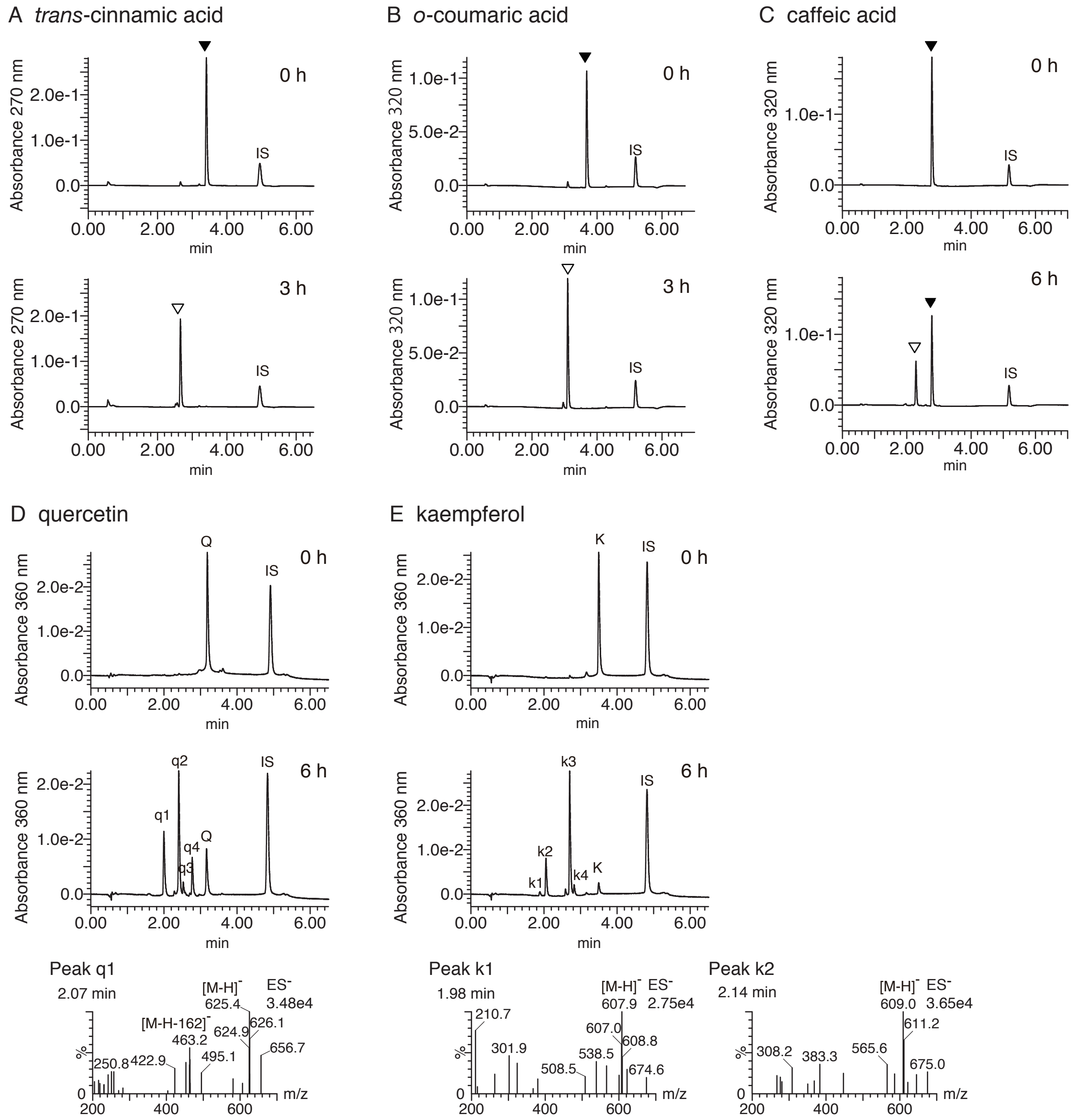

Supplementary Figure S6 Bioconversion of cinnamates and flavonols by the Ec-IbGT1 system.

UPLC profiles of the culture media at 0 h and 3 or 6 h after the addition of *p*-coumaric acid (A), *o*-coumaric acid (B), caffeic acid (C), quercetin (D), and kaempferol (E). The products were separated using LC-method A. The MS spectra corresponding to the products (peaks q1, k1, and k2) were shown below the chromatogram. Peak identification: open triangles, product; closed triangles, substrate; Q, quercetin; q1, quercetin diglucoside; q2, quercetin 7-*O*-glucoside; q3, quercetin 3-*O*-glucoside; q4, quercetin glucoside; K, kaempferol; k1-k2, kaempferol diglucoside; k3, kaempferol 7-*O*-glucoside; and k4, kaempferol 3-*O*-glucoside. IS: Internal standard (chrysin).

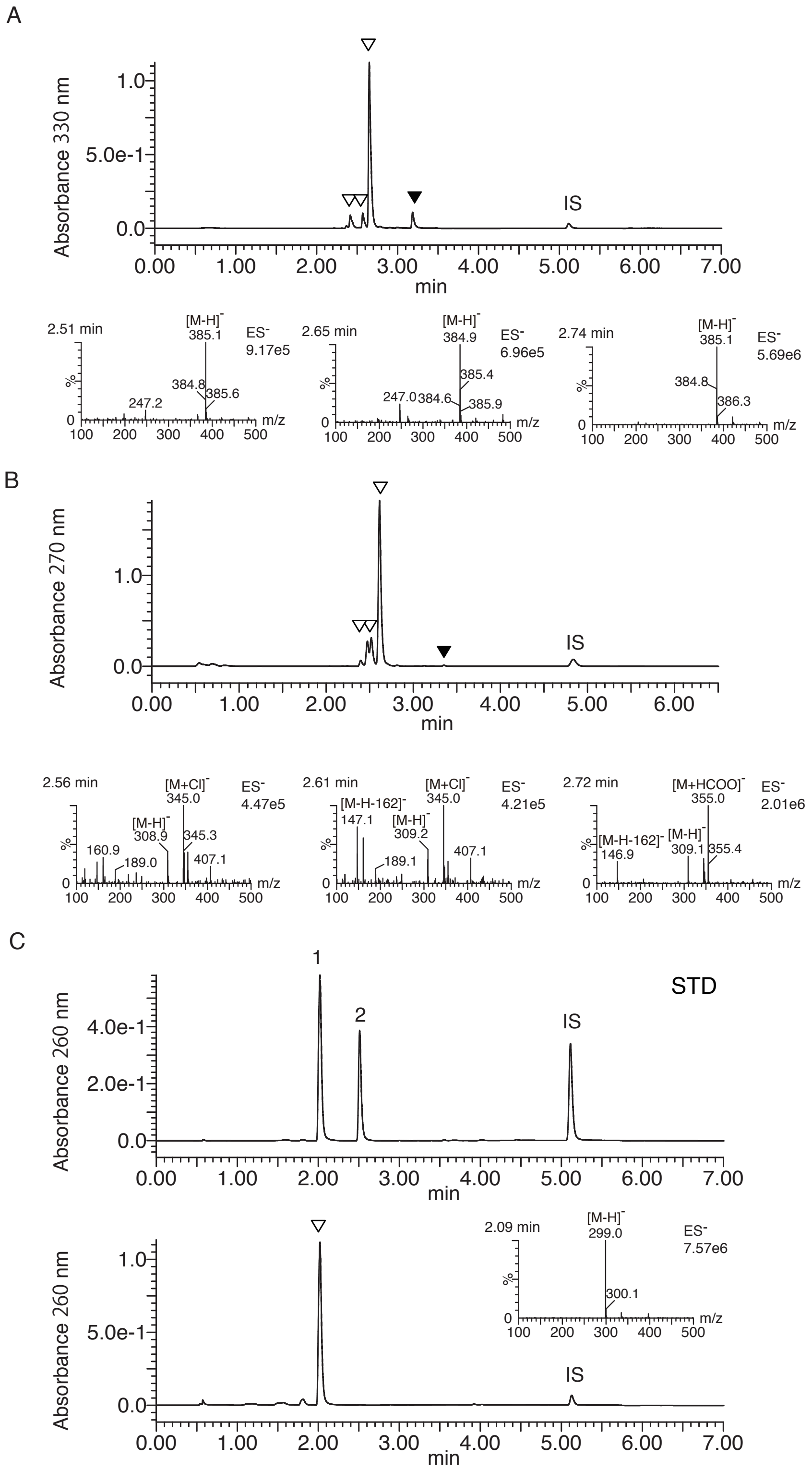

Supplementary Figure S7 Results of the scaled-up bioconversion by Ec-IbGT1 after repeated administration of substrates.

A–B, Results of bioconversion of sinapic acid (A) and *trans*-cinnamic acid (B) after 30 h of initiation. Open and closed triangles indicate products and remaining substrate, respectively. MS spectra of each product are shown in the lower panel. C, Results of *p*-hydroxybenzoic acid bioconversion. Upper panel: standard compounds (STD). Lower panel: results of bioconversion after 20 h of initiation. The MS spectrum of the product is shown in the panel. Peak identification: 1 and open triangle; *p*-hydroxybenzoly glucose (product); 2, *p*-hydroxybenzoic acid; IS, internal standard (chrysin).

A

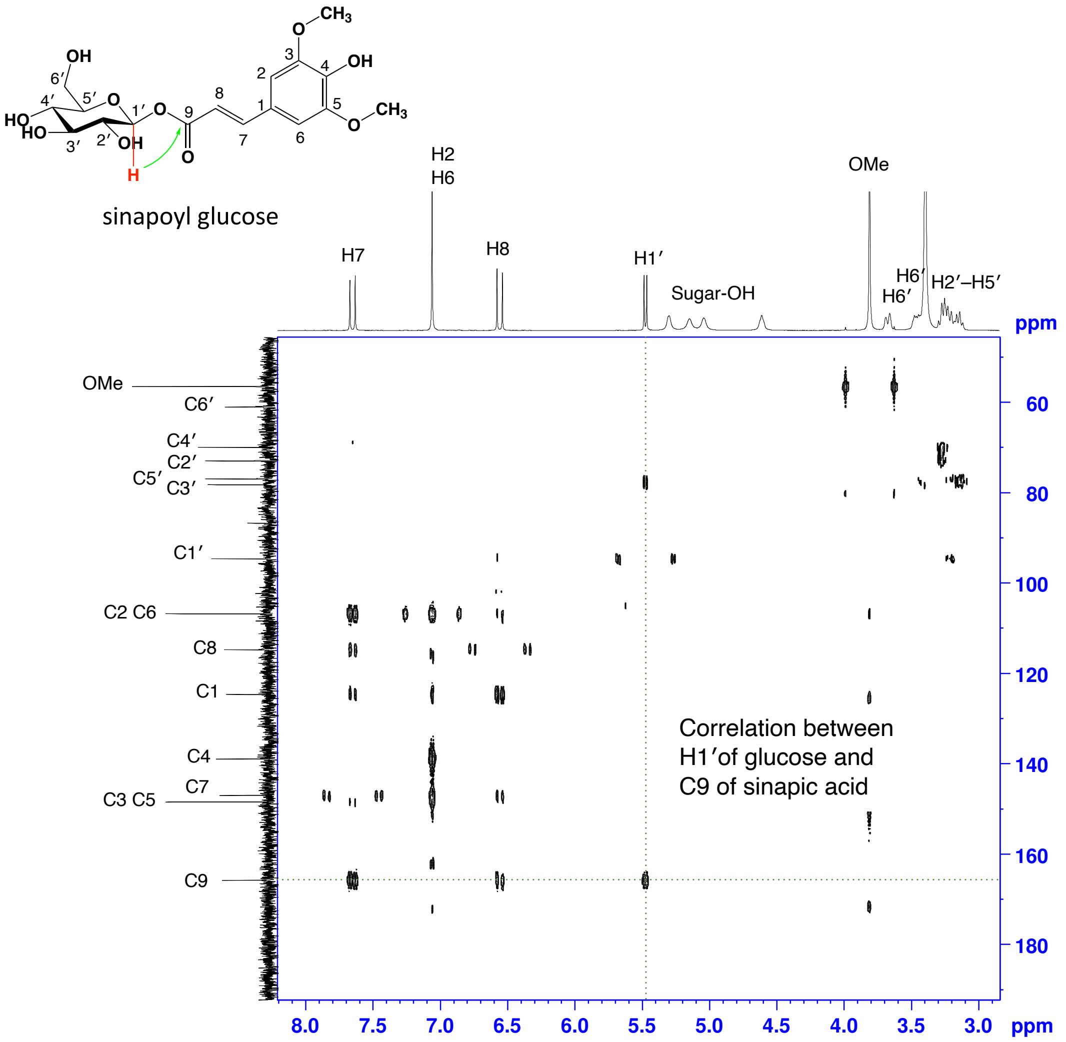

Supplementary Figure S8

B

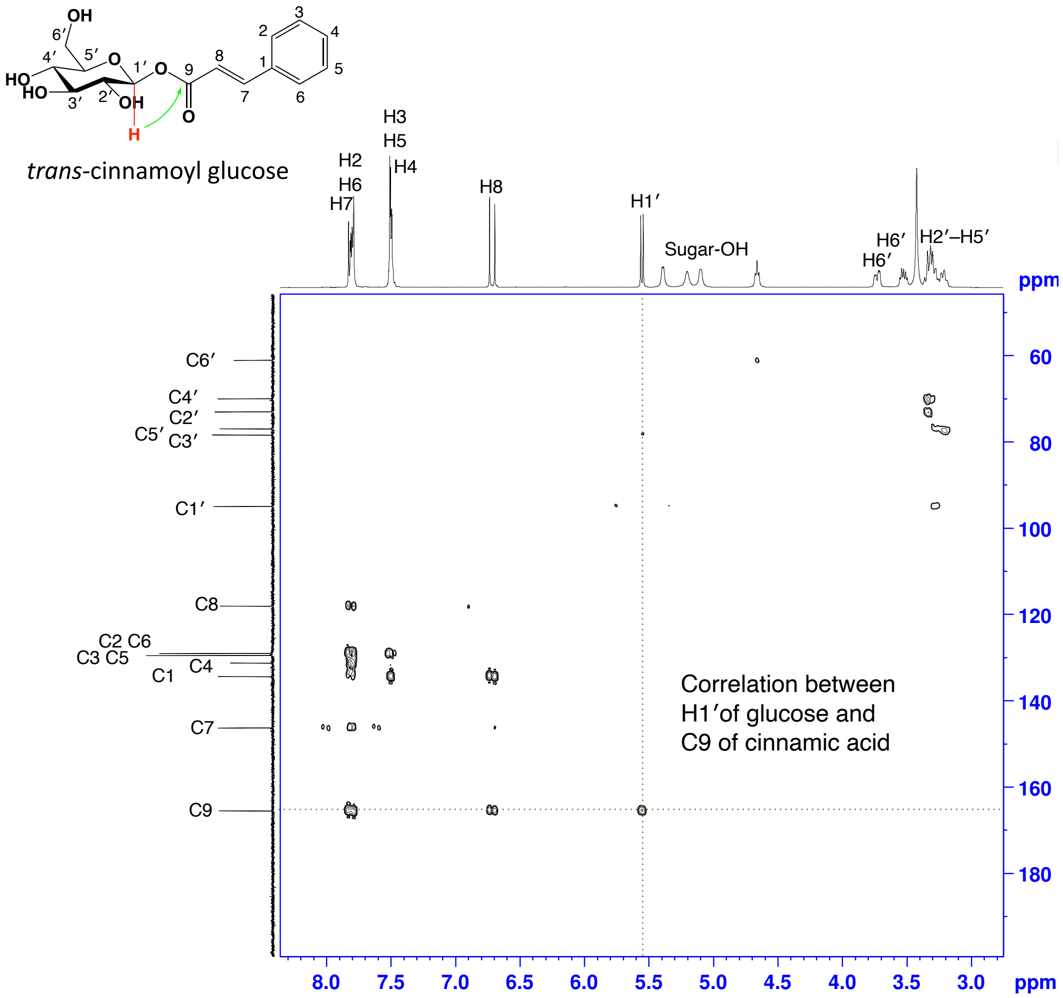

Supplementary Figure S8 Heteronuclear multiple bond correlation (HMBC) analysis of the compounds produced by bioconversion using Ec-IbGT1 system.

The purified product was dissolved in dimethyl sulfoxide (DMSO)-d_6 and analyzed by NMR. Each panel shows the result of HMBC analysis of sinapoyl glucose (A) and *trans*-cinnamoyl glucose (B). Green arrow in chemical structure indicates the HMBC correlation between H-1′ of glucose and carbon of sinapic acid or *trans*-cinnamic acid.

A

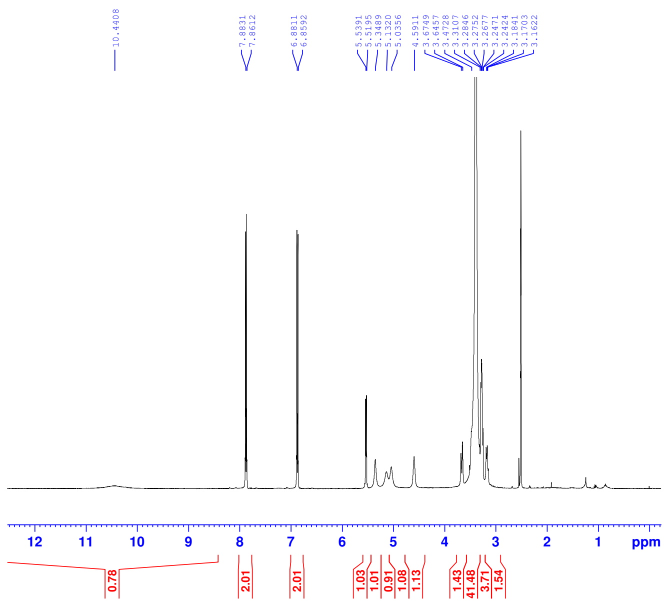

B

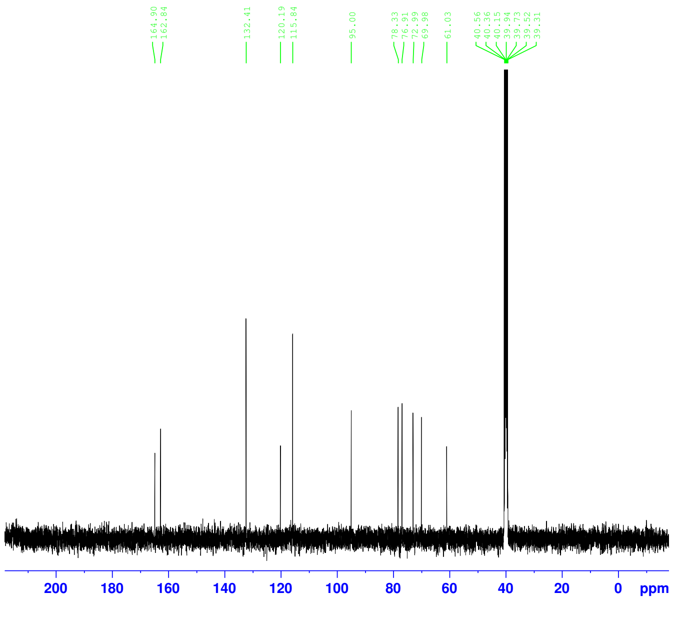

Supplementary Figure S9 NMR analysis of *p*-hydroxybenzoyl glucose produced by bioconversion using Ec-IbGT1.

Purified *p*-hydroxybenzoyl glucose was dissolved in dimethyl sulfoxide (DMSO)-d_6 and analyzed by NMR spectroscopy. A, ^{1}H-NMR, B, ^{13}C-NMR.
